# Radar/INS Integration and Map Matching for Land Vehicle Navigation in Urban Environments

**DOI:** 10.3390/s23115119

**Published:** 2023-05-27

**Authors:** Mohamed Elkholy, Mohamed Elsheikh, Naser El-Sheimy

**Affiliations:** 1 Department of Geomatics Engineering, University of Calgary, Calgary, AB T2N 1N4, Canada; mohamed.elkholy@ucalgary.ca (M.E.); elsheimy@ucalgary.ca (N.E.-S.); 2 Department of Transportation Engineering, Alexandria University, Alexandria 21544, Egypt; 3 Electronics and Electrical Communication Engineering Department, Tanta University, Tanta 31512, Egypt

**Keywords:** radar/INS integration, radar odometry, FMCW radar, map matching, vehicle navigation, GNSS-challenging environments, GNSS outages, urban navigation

## Abstract

Autonomous navigation requires multi-sensor fusion to achieve a high level of accuracy in different environments. Global navigation satellite system (GNSS) receivers are the main components in most navigation systems. However, GNSS signals are subject to blockage and multipath effects in challenging areas, e.g., tunnels, underground parking, and downtown or urban areas. Therefore, different sensors, such as inertial navigation systems (INSs) and radar, can be used to compensate for GNSS signal deterioration and to meet continuity requirements. In this paper, a novel algorithm was applied to improve land vehicle navigation in GNSS-challenging environments through radar/INS integration and map matching. Four radar units were utilized in this work. Two units were used to estimate the vehicle’s forward velocity, and the four units were used together to estimate the vehicle’s position. The integrated solution was estimated in two steps. First, the radar solution was fused with an INS through an extended Kalman filter (EKF). Second, map matching was used to correct the radar/INS integrated position using OpenStreetMap (OSM). The developed algorithm was evaluated using real data collected in Calgary’s urban area and downtown Toronto. The results show the efficiency of the proposed method, which had a horizontal position RMS error percentage of less than 1% of the distance traveled for three minutes of a simulated GNSS outage.

## 1. Introduction

Autonomous navigation relies on a global navigation satellite system (GNSS) to estimate the vehicle’s location in open-sky and suburban environments [1]. However, relying only on the GNSS is inadequate because the GNSS signal can be affected by the surrounding environment. For example, the GNSS signal will be blocked in underground and indoor areas. Moreover, the GNSS signal suffers from multipath errors and loss of lock in GNSS-challenging areas such as downtown [2]. On the other hand, an inertial navigation system (INS) can accurately estimate the vehicle’s relative position, velocity, and attitude but only for a short time, as the INS solution deteriorates with time [3]. Therefore, GNSS/INS integration is employed in various navigation applications to overcome the limitations of both the GNSS and INS. There are two major types of GNSS/INS integration: loosely coupled integration [4] and tightly coupled integration [5]. 

Although GNSS/INS integration estimates the vehicle’s navigation states with reliable accuracy in the short term, the accuracy will deteriorate during long GNSS outages. During these outages, the system relies only on inertial measurement unit (IMU) sensors (accelerometers and gyroscopes) whose output drifts rapidly with time, especially when utilizing low-cost IMUs [6]. Therefore, in autonomous navigation systems, more sensors, such as vehicle onboard sensors, lidar, vision sensors, and radar, are required to aid the GNSS/INS system. However, each of these sensors has its advantages and disadvantages.

Gao et al. [7] used a vehicle’s chassis sensors to compensate for the GNSS outage. Wheel speed sensors were used to estimate the vehicle’s forward velocity, while the lateral velocity was estimated using the steering wheel angle sensor. The fusion of the kinematic model with the estimated velocity from the onboard sensors improved the accuracy of the vehicle localization during GNSS signal outages. 

Lidar has mainly been used to percept the surrounding environment [8]. Recently, lidar has been used to aid IMUs in GNSS-denied environments and for simultaneous localization and mapping (SLAM) [9,10,11]. However, lidar is affected by snowy weather and requires a high level of computational power. Moreover, lidar is a high-cost sensor. On the other hand, a vision sensor is mainly used to detect highway lanes and objects in the surrounding environment, and it can be fused with an INS to limit the IMU’s errors [12,13]. Nevertheless, the drawbacks of vision sensors are that they cannot work under different light conditions and are affected by rain, fog, and snowy weather. An odometer can also aid the INS in autonomous navigation applications by providing the vehicle’s forward velocity; however, it is affected by slippery roads, an unequal diameter, and unequal pressure [14]. 

In contrast, radar is a low-cost sensor that works in different weather and light conditions. Thus, it is known as an all-weather sensor. Therefore, the research in this paper is based on the fusion of radar and an INS in environments that are challenging for a GNSS. Furthermore, the motion constraints of a land vehicle [15] are also applied for better performance, e.g., the non-holonomic constraint (NHC) [15], zero-velocity update (ZUPT) [16], and the zero-integrated heading rate (ZIHR) [16].

Radar is mainly used in adaptive cruise control (ACC) to detect the ranges and relative speeds of surrounding vehicles and objects to make autonomous driving safe. 

There are two types of radar: 360° radar and static radar. The 360° radar rotates 360 degrees to scan the surrounding environment. It is known as imaging radar since it returns only the range, azimuth, and density value for each detected object. In contrast, static radar does not rotate and has a limited field of view. There are two types of static radar. The first type, continuous wave (CW) radar, can measure the Doppler velocity between an object and the radar unit, whereas the second type can measure the range and the Doppler velocity of the objects; this type is known as frequency modulated CW (FMCW) radar. Both 360° radar and static radar are used in autonomous navigation applications. 

Elkholy et al. [17,18] applied the oriented fast and rotated brief (ORB) technique to detect and match the objects from 360° radar frames to estimate the transition and rotation between every two frames. The estimated solution was integrated with an IMU through an EKF to improve the navigation solution. In Elkholy et al. [19], FMCW radar was utilized, and a novel algorithm was adopted to estimate the vehicle’s relative position and rotation. With the knowledge of the vehicle’s average speed and radar data rate, the change in the range and azimuth between the objects in any two successive radar frames can be estimated. Then, the corresponding objects can be determined. The corresponding points were used to estimate the rotation and transition between any two radar frames. Then, the radar solution was integrated with an IMU for an ego-motion estimation. Rashed et al. [20] used an FMCW radar fixed at the center of the front bumper to estimate the distance traveled. The radar output was integrated with a reduced inertial sensor system (RISS). A RISS employs only three sensors (one vertical gyroscope and two horizontal accelerometers), whereas the full IMU contains three gyroscopes and three accelerometers. In Abosekeen et al. [21], the FMCW radar was fixed facing the ground to estimate the vehicle’s forward speed from the Doppler frequency of the reflected radar waves. A RISS solution was integrated with an IMU through an EKF to improve the navigation solution in GNSS-denied environments. 

The iterative closest point (ICP) method is the most commonly used method for estimating the relative transition and rotation between any two radar frames by minimizing the distance between point clouds [22,23,24]. Normal distribution transform (NDT) is another technique that relies on building a normal distribution model for point clouds in every radar frame. These models are then matched to estimate the vehicle’s ego-motion [25]. 

To improve the accuracy of the vehicle’s position, a predefined map was utilized to correct the integrated solution by matching the integrated position with map links. The predefined map can be generated using sensors such as cameras, lidar, or radar [26]. However, using these sensors to make the maps is time-consuming due to the high computational cost and the high volume of data to be stored. The best solution is to use navigation maps such as Google Maps or OpenStreetMap (OSM) [27]. OSM was created by volunteers using standard surveying techniques. OSM provides rich information, including road links, buildings, traffic signs, the number of lanes, and other data. Radar frames can be matched with predefined digital maps to estimate the vehicle’s transition and rotation [28]. 

The first step in map matching is to determine the vehicle’s location on the map. In other words, to select the correct map link where the position will be projected. Different algorithms can be utilized to determine this map link. Geometrical and topological techniques depend on finding the distances between the position and the map links, and then the map link with the minimum distance will be selected [29]. The second technique is the probabilistic algorithm. This algorithm draws a confidence ellipse from the position’s standard deviation and determines which map links intersect with this ellipse [30]. Another technique to determine the map link is to use the fuzzy logic algorithm. The fuzzy logic algorithm takes the inputs from the navigation sensors and returns the map links as likelihoods [31]. The main difference between the previous techniques is the method of selecting the correct map link for matching.

This research focuses on radar and INS integration to meet the continuity requirements, compensate for GNSS outages, and improve the navigation solution for land vehicles. Moreover, the integrated solution is corrected by applying map matching through open access OSM. Section 2 describes the methodology and the integration scheme used in this research. Section 3 shows the experimental work and the results from the proposed method. Finally, Section 4 concludes the presented work.

## 2. Methodology

**Figure 1:**
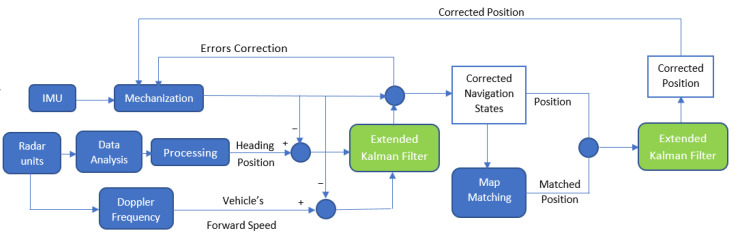
Flow chart of the developed radar/INS/map-matching integration technique during GNSS outage.

The navigation system proposed in this research relies on four FMCW radar units, an IMU, and OpenStreetMap (OSM). The four radar units were used for the ego-motion estimation, while two of the four units were used to estimate the vehicle’s forward speed. The map-matching technique was applied at the end to correct the integrated position to OSM. A successive EKF was implemented to correct the navigation solution and limit the IMU errors. Figure 1 shows a block diagram of the proposed method. The next subsections describe the techniques applied to use radar for an ego-motion estimation, to estimate the vehicle’s forward velocity, in the EKF prediction and update stages, and in map matching.

### 2.1. Radar for Ego-Motion Estimation

The four static radar units were fixed on the roof of the vehicle to collect and detect the objects around the vehicle. The four frames from all the radar units were synchronized and combined into one frame representing the environment around the vehicle. Thus, the four static radar units worked as a 360° radar unit. 

Before processing the radar data, noisy data and outliers were detected and removed by applying a set of constraints. First, the close and far points were removed because these points were considered ghost points. The Doppler frequency was also used to differentiate between static and moving objects. The moving objects were removed from the radar frames. Finally, a threshold was applied to remove the outliers with intensity values lower than the threshold.

After the data analysis and preprocessing, data association was applied to find the corresponding points between radar frames. The change in the range and azimuth between any two corresponding points in two successive radar frames could be estimated based on the knowledge of the radar data rate (about 8 Hz) and the average vehicle speed. 

**Figure 2:**
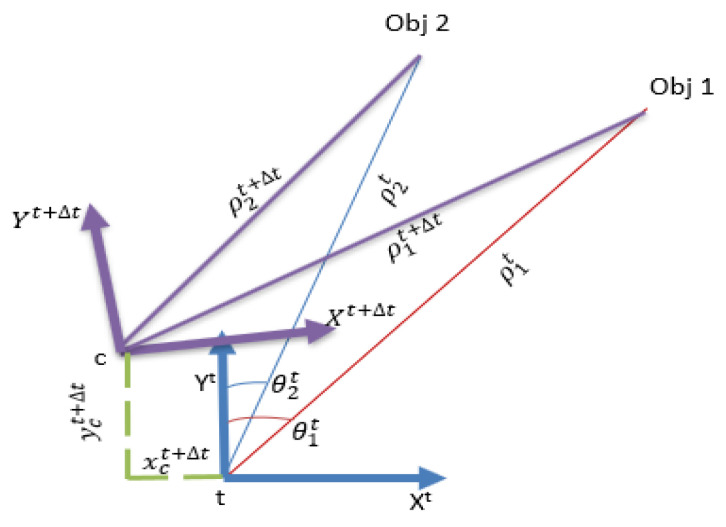
The proposed method for estimating the vehicle’s ego-motion.

A novel algorithm was implemented to estimate the vehicle’s ego-motion (Figure 2). This algorithm calculates the coordinates of the objects ($x_{i}^{t},\;y_{i}^{t})\;$in the first radar frame. It then uses these calculated coordinates and the range in the second frame to estimate the coordinates of the radar center ($x_{c}^{t+\Delta t}\;$, $y_{c}^{t+\Delta t}$). The difference between the calculated radar center in the second frame and the one in the first frame represents the transition between the two frames.

(1) $$x_{i}^{t}=\rho_{i}^{t}\sin\left(\theta_{i}^{t}\right)$$

(2) $$y_{i}^{t}=\rho_{i}^{t}\cos\left(\theta_{i}^{t}\right)$$

(3) $$\;\rho_{i}^{t+\Delta t}=\sqrt{{\left(x_{i}^{t}-x_{c}^{t+\Delta t}\right)}^{2}+{\left(y_{i}^{t}-y_{c}^{t+\Delta t}\right)}^{2}}$$

The rotation between the two frames can be estimated by calculating the difference between the bearings of the objects at a time (t) and time (t+Δt).

(4) $$\theta_{t,t+\Delta t}=\theta_{i}^{t}-\theta_{i}^{t+\Delta t}$$

If we have multiple corresponding objects between any two radar frames, the least squares method is applied to obtain the final transformation and rotation between the two frames.

(5) $$\delta x=-{\left(A^{T}C_{l}^{-1}A\right)}^{-1}\left(A^{T}C_{l}^{-1}W\right)$$

where δx = is the vector containing the corrections for the transition (*dx* and *dy*) and the rotation (θ) between any two radar frames.

A = the design matrix.

$C_{l}$ = the measurement variance-covariance matrix.

W = the misclosure vector.

### 2.2. Estimating the Vehicle’s Forward Speed

FMCW radar provides information about the Doppler frequency between the radar unit and the detected objects. Therefore, the relative velocity between the radar and the objects can be estimated from the Doppler frequency. This relative velocity will be the vehicle’s forward velocity if the detected objects are static. 

There were four radar units fixed on the roof of the vehicle. The two radar units at the front right and left of the vehicle were chosen to estimate the vehicle’s forward velocity. Then, the average forward velocity was calculated from the two units. The radar provides the relative velocity of each object based on the Doppler frequency. The relative velocity value could be positive if the objects are moving far from the vehicle and negative if the objects are coming toward the vehicle. The vehicle velocity estimation depends on the static objects, while the moving objects act as outliers and should be excluded. The static objects will appear to approach the vehicle through the two front radar units. Therefore, only objects with a negative relative velocity were kept to exclude the objects moving away from the vehicle.

**Figure 3:**
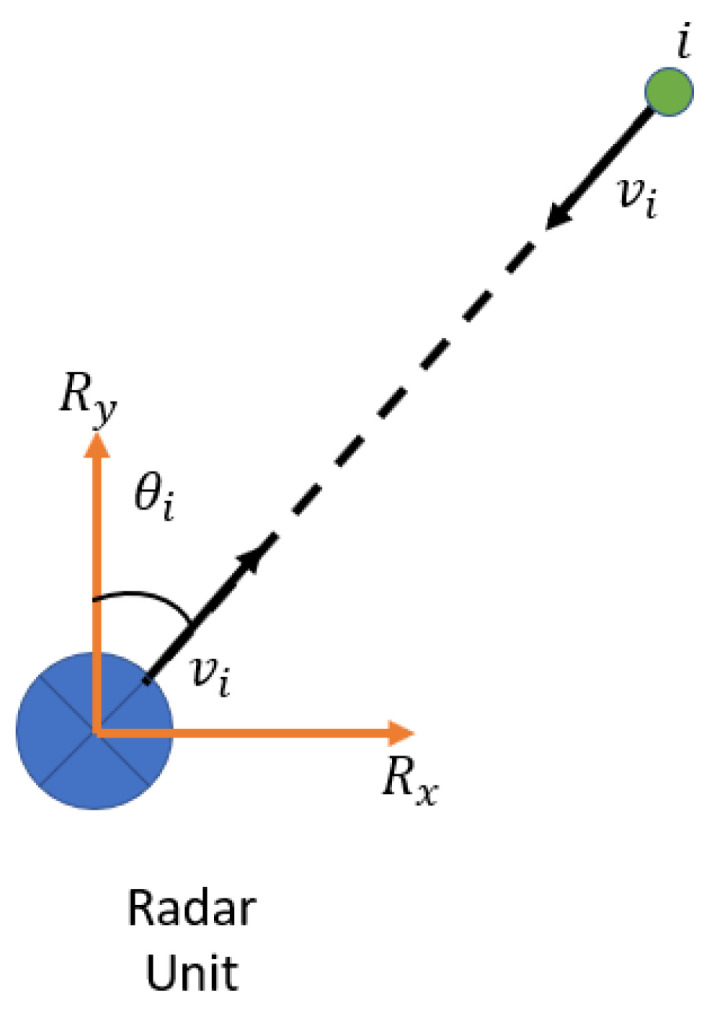
The relative motion between the radar unit and a static object (i).

If a static object (i) is detected by a radar unit, as shown in Figure 3, the relative velocity between this object and the radar ($v_{i})$ will represent the absolute radar velocity ($v_{R})$ since the static object’s velocity is zero. Using the bearing angle ($\theta_{i}$), the radar velocity can be decomposed into the x and y components in the radar frame using the equation:

(6) $$v_{i}=v_{R}=v_{Rx}*\sin\left(\theta_{i}\right)+v_{Ry}\cos\left(\theta_{i}\right)$$

where $v_{Rx}$ and $v_{Ry}$ are the radar velocity components in the radar frame (x and y). 

Suppose there are multiple objects (n) in the radar frame. Then, the radar velocities for x and y in radar local frame can be estimated. The least squares estimation is applied to estimate a reliable solution.

(7) $$\left[\begin{array}{c} v_{1}\\ \vdots \\ v_{n} \end{array}\right]=\left[\begin{array}{cc} \sin\left(\theta_{1}\right) & \cos\left(\theta_{1}\right)\\ \vdots & \vdots \\ \sin\left(\theta_{n}\right) & \cos\left(\theta_{n}\right) \end{array}\right]\left[\begin{array}{c} v_{Rx}\\ v_{Ry} \end{array}\right]$$

Now, the magnitude ($V_{R}$) and the orientation angle (α) can be calculated from $v_{Rx}$ and $v_{Ry}$.

(8) $$V_{R}=\sqrt{{\left(v_{Rx}\right)}^{2}+{\left(v_{Ry}\right)}^{2}}$$

(9) $$\alpha =\tan^{-1}\frac{v_{Rx}}{v_{Ry}}$$

The final step is to convert the radar velocity ($V_{R}$) from the radar frame into the vehicle frame to estimate the vehicle’s forward speed ($V_{veh}$).

(10) $$V_{veh}=V_{R}\cos\left(\alpha +\varphi \right)$$

where φ is the boresight angle between the radar and vehicle frames.

Since the IMU body frame was aligned with the vehicle frame, the vehicle’s estimated forward speed is used to estimate the vehicle’s transition in the east and north directions. Then, the updated position of the vehicle can be estimated.

(11) $$\Delta y_{t}=V_{veh}\Delta t\cos\left(az_{t}\right)$$

(12) $$\Delta x_{t}=V_{veh}\Delta t\sin\left(az_{t}\right)$$

where $\Delta x_{t}$ and $\Delta y_{t}$ are the transitions in the east and north directions at time t, Δt is the time difference between the radar frames, and $az_{t}$ is the heading angle at time t.

### 2.3. Radar/INS Integration

In open-sky environments, loosely coupled GNSS/INS integration was used to estimate the vehicle’s navigation solution. However, a successive Kalman filter was implemented in case of a GNSS outage. First, the radar solution was integrated with the INS solution through a closed-loop EKF. Then, the corrected position, obtained via map matching, was also integrated through an EKF to aid the IMU and overcome long-time errors. One of the practical aspects of multi-sensor fusion is data synchronization, which can be achieved in various ways, such as interpolation or by searching for the closest values [32]. In our work, due to the high IMU rate compared to the radar rate, the algorithm searched for the closest IMU timestamp for each radar frame. 

A KF consists of two phases (the prediction stage and the update stage). There are two main models needed for the KF. The system model is the INS model, and it is the core for the prediction stage to predict the error states and the covariance matrix. The other model is the observation model, which depends on radar to update the navigation filter to estimate the error state to correct the navigation solution. There are 15 error states (δx).

(13) $$\delta x_{15x1}={\left[\begin{array}{ccccc} \delta p_{1x3} & \delta v_{1x3} & \delta \theta_{1x3} & \delta b_{a1x3} & \delta b_{g1x3} \end{array}\right]}^{T}$$

where $\delta p_{1x3}$ is the position error, $\delta v_{1x3}$ is the velocity error, $\delta \theta_{1x3}$ is the attitude error, $\delta b_{a1x3}$ is the accelerometer bias, and $\delta b_{g1x3}$ is the gyroscope bias. 

The system model in the continuous case is described as follows:

(14) $$\dot{x}=Fx+Gw$$

where $\dot{x}$ is the time rate of change of the state vector, F is the dynamic matrix, x is the state vector, G is the noise coefficient matrix, and w is the system noise.

The system model in the discrete case is as follows:

(15) $$x_{t+\Delta t}^{\prime }=\emptyset_{t,t+\Delta t}x_{t}+w_{t}$$

(16) $$\emptyset_{t,t+\Delta t}=I+F\Delta t+1/2F^{2}\Delta t^{2}$$

(17) $$p_{t+\Delta t}^{\prime }=\emptyset_{t,t+\Delta t}p_{t}\emptyset_{t,t+\Delta t}^{T}+Q_{t}$$

where $x_{t+\Delta t}^{\prime }$ is the predicted state vector at the time (t+Δt), ∅ is the transition matrix, $x_{t}$ is the state vector at the time (t), $p_{t+\Delta t}^{\prime }$ is the predicted covariance matrix at the time (t+Δt), $p_{t}$ is the covariance matrix at the time (t), $w_{t}$ is the system noise at the time (t), and $Q_{t}$ is the process noise matrix.

The observation model is the core of the update stage, and it depends on the other available sensors to aid the IMU; in this case, the estimated position and heading from the radar were used to build the observation model.

(18) z=Hx+η

where z represents the measurements, H represents the design matrix, and η represents the measurement noise.

The updated stage is as follows:

(19) $$K_{t+\Delta t}=p_{t+\Delta t}^{\prime }H_{t+\Delta t}^{T}{\left(H_{t+\Delta t}p_{t+\Delta t}^{\prime }H_{t+\Delta t}^{T}+R_{t+\Delta t}\right)}^{-1}$$

(20) $${\hat{x}}_{t+\Delta t}=x_{t+\Delta t}^{\prime }+K_{t+\Delta t}\left(Z_{t+\Delta t}-H_{t+\Delta t}x_{t+\Delta t}^{\prime }\right)$$

(21) $${\hat{p}}_{t+\Delta t}=[I-K_{t+\Delta t}H_{t+\Delta t}]\;p_{t+\Delta t}^{\prime }$$

where $K_{t+\Delta t}\;$is the gain matrix, $R_{t+\Delta t}$ is the covariance matrix of the measurement noise, ${\hat{x}}_{t+\Delta t}\;$is the updated state vector, and ${\hat{p}}_{t+\Delta t}$ is the updated covariance matrix of the state vector.

### 2.4. Map Matching

**Figure 4:**
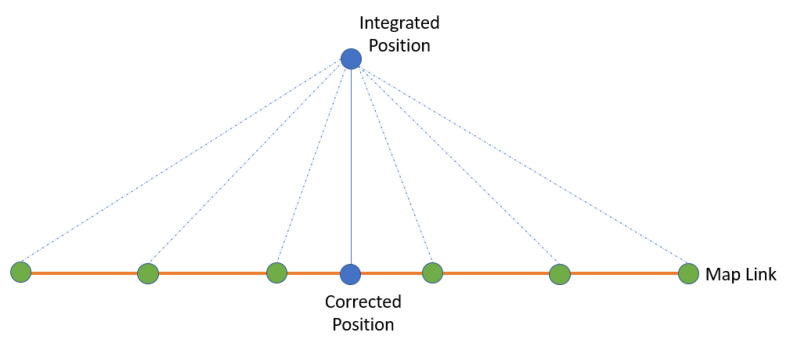
Map-matching technique.

OSM is an open access map anyone can download, edit, or update. Maps of Calgary and Toronto were downloaded and employed in this research. The map-matching technique was applied in two steps. The first step was to select the correct map link. Geometrical and topological techniques were exploited to determine the correct map link. A map link consists of points or nodes. The distance between the integrated position and the nodes was calculated (Figure 4), and the map link with the minimum distance was selected. Another constraint that can be adapted to ensure the selected map link is correct is the azimuth. The integrated azimuth should be equal to the map link azimuth. 

**Figure 5:**
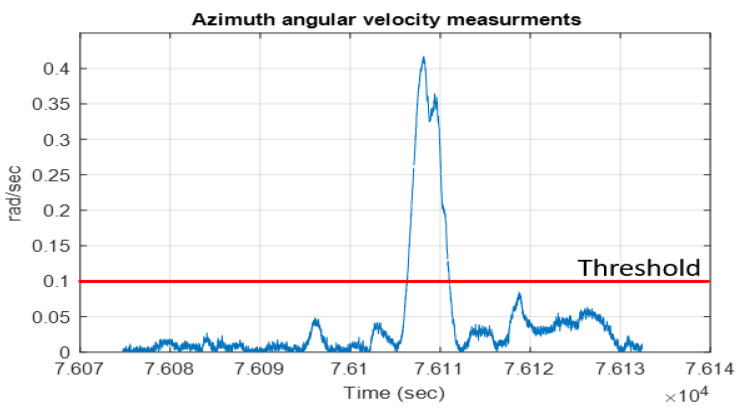
Angular velocity measurements.

The second step was to project the integrated position onto the selected map link to find the vehicle’s corrected position. These two steps were implemented each time unless the vehicle began to turn. This turn could be detected by checking the azimuth angular velocity measurements. If the absolute azimuth angular velocity measurements were higher than a threshold, it meant that the vehicle had reached an intersection and was making a turn. Moreover, the azimuth angular velocity measurements could be utilized to determine if the vehicle was turning right or left, which aided in selecting the new map link (Figure 5).

## 3. Experimental Work and Results

**Figure 6:**
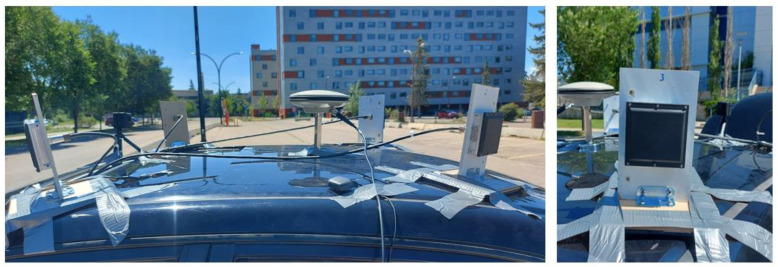
The four UMRR-11 Type 132 radar units used to collect the data in Calgary.

**Figure 7:**
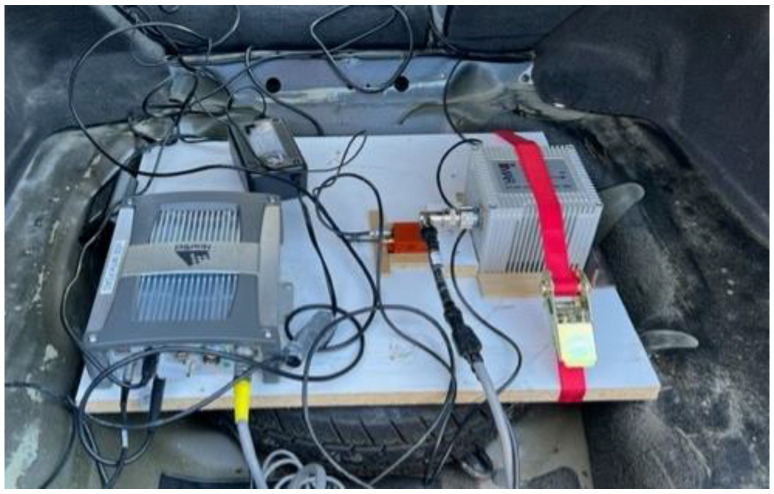
The reference system and the Xsens unit in the Calgary test.

Two real datasets were collected. The first one was collected in a suburban area in Calgary, Alberta, Canada, while the second data was collected in downtown Toronto, Ontario, Canada. For the Calgary dataset, four FMCW UMRR-11 Type 132 radar units were mounted on the vehicle’s roof [33]. In addition, the Xsens MTi-G-710 module was used to collect the IMU and GNSS measurements [34], and the reference data were collected by a Novatel SPAN-SE system with an IMU-FSAS (Figure 6 and Figure 7). On the other hand, in the Toronto test, four FMCW UMRR-96 Type 153 radar units were used [35]. In addition, the u-blox ZED-F9R module was used to collect the IMU and GNSS measurements [36]. Finally, the reference data were collected by a Novatel PwrPak7 system with an IMU-KVH1750.

The first algorithm applied was to estimate the vehicle’s forward velocity from two of the four radar units. Data from the two radar units at the front right and left of the vehicle were utilized for this purpose. Then, radar odometry was applied to the four radar units. Finally, the radar solution was integrated with the INS, and map matching was applied to the integrated position. The next subsections show the results for the two datasets. 

### 3.1. Calgary Data

**Figure 8:**
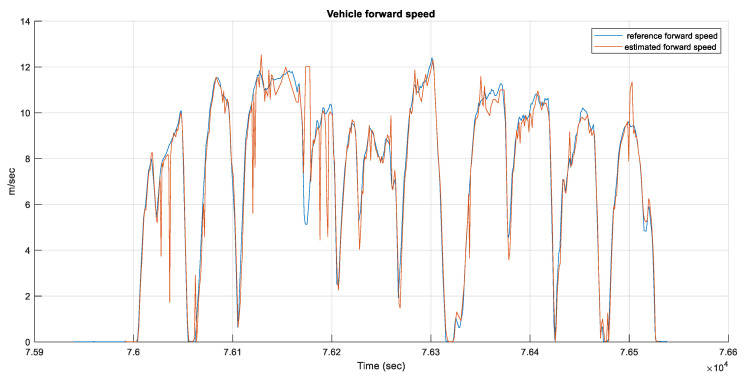
The estimated average forward speed from the radar unit versus the reference forward speed for the Calgary data.

For the Calgary data, Figure 8 shows the estimated average forward velocity from the two radar units compared to the forward velocity from the reference system. The RMSE for the estimated average forward velocity is 1.08 m/s, the distance traveled was 2.1 km, and the test duration was 9.98 min.

**Figure 9:**
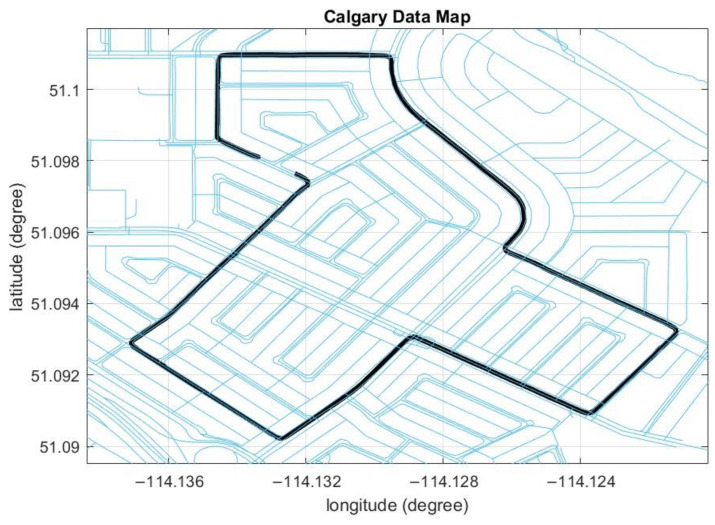
The OSM for Calgary data. The black line is the reference trajectory.

**Figure 10:**
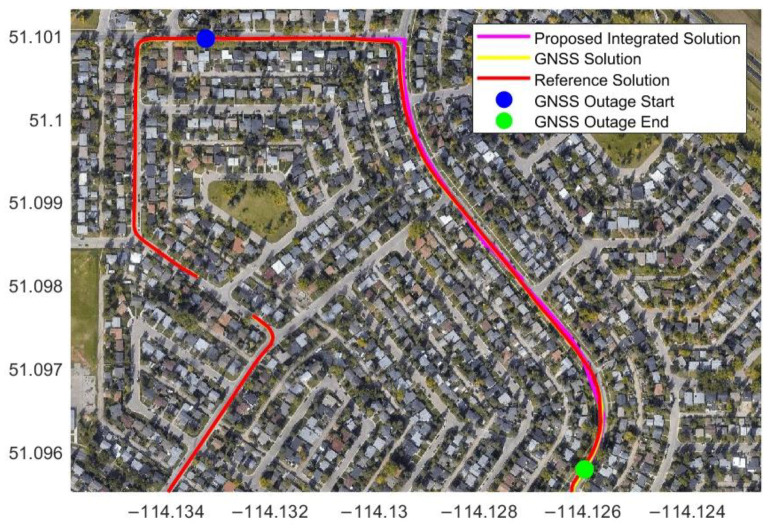
The estimated trajectory from radar/INS integration with map matching during a 90 s simulated GNSS outage in Calgary data.

The map on which the data was collected was downloaded from the OSM website for map matching (Figure 9). A simulated GNSS outage was introduced for 90 s, and the corrected position after applying the map-matching technique is shown in Figure 10. 

Table 1 shows the position RMSE from the radar/INS integration through a closed-loop EKF with map matching during four simulated GNSS signal outages lasting 30 to 90 s. The position RMSE values are 2.69 m and 22.89 m for the 30 s and 90 s GNSS outages, respectively, while the percentage errors are about 1 % and 2.46 % for the traveled distances of 259.34 m and 929.82 m, respectively. These results indicate that applying the map-matching technique corrected the vehicle’s integrated position since the integrated position was projected onto the nearest point on the map lines. Therefore, the projected position was close to the reference position. 

### 3.2. Toronto Data

**Figure 11:**
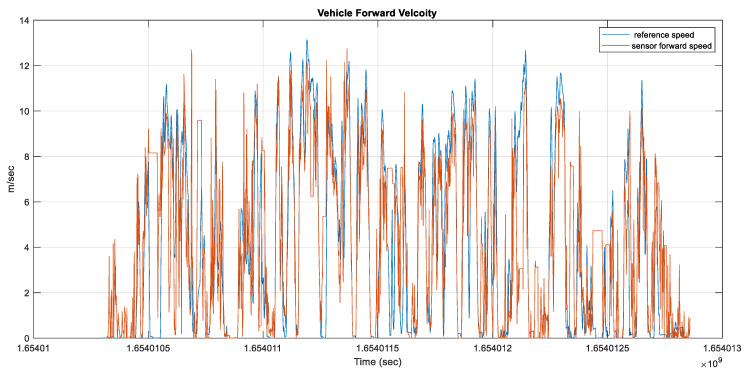
The estimated average forward speed from the radar unit versus the forward reference speed for Toronto data.

The vehicle’s forward velocity was estimated, as shown in Figure 11. The RMSE for the estimated average forward velocity is 2.65 m/s, the distance traveled was 9.94 km, and the test duration was 42.7 min.

**Figure 12:**
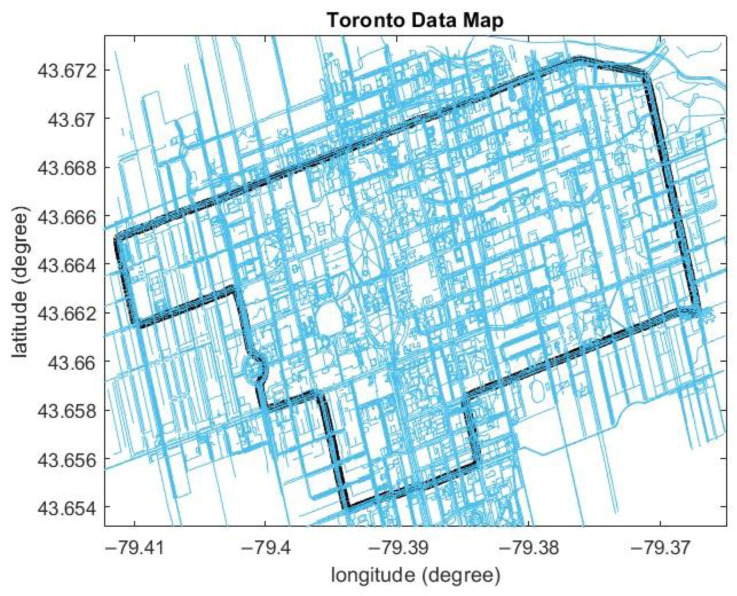
The OSM for Toronto data. The black line is the reference trajectory.

Two different simulated GNSS signal outages were introduced to prove the efficiency of the proposed algorithm. The simulated outage duration ranged from 30 s to 180 s. Finally, the road map was downloaded to apply the map-matching technique (Figure 12).

**Figure 13:**
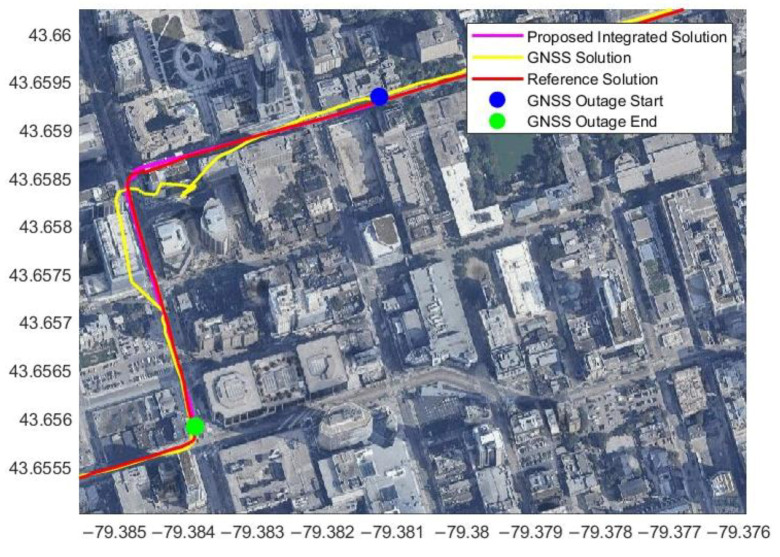
The estimated trajectory from radar/INS integration with map matching during three minutes of a simulated GNSS signal outage in Toronto data.

Since these data were collected in the downtown area, the GNSS signal suffered from blockage and multipath errors. However, Figure 13 shows that the radar/INS integrated solution with map matching is better than the GNSS solution, demonstrating the benefit of the proposed integrated solution. 

From Table 2, the position percentage error is less than 0.5% for the three minutes of the simulated GNSS outage. The position RMSE is 2.72 m, and the distance traveled was 611.69 m for the three-minute outage (Figure 13). The percentage errors are 0.61%, 0.86%, and 1.01% for the 60 s, 90 s, and 120 s GNSS outages, respectively, while the traveled distance ranged from 204 m to 304 m. 

**Figure 14:**
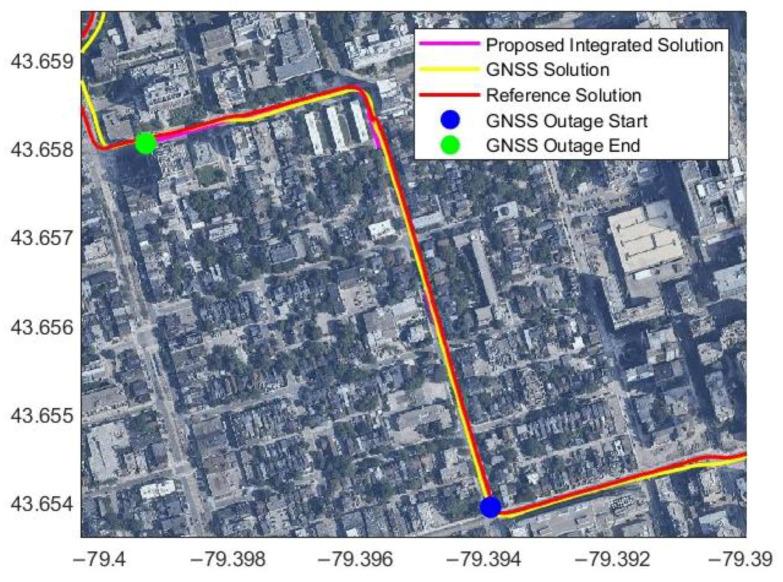
Another example of the estimated trajectory from radar/INS integration with map matching during three minutes of a simulated GNSS signal outage using Toronto data.

Figure 14 shows the integrated solution with map matching during another simulated GNSS outage. Table 3 shows the results for the position error for this GNSS simulated outage. The GNSS outage duration was from 30 s to 180 s. The position RMSE is 4.47 m for a traveled distance of 845.74 m, and the percentage error during the three-minute GNSS signal outage is 0.53%. For 60 s, 90 s, and 120 s of GNSS outage, the percentage errors are 0.71%, 0.64%, and 0.61%, respectively, and the traveled distance was from 495.16 m to 679.49 m.

### 3.3. Enable and Disable Map Matching

**Figure 15:**
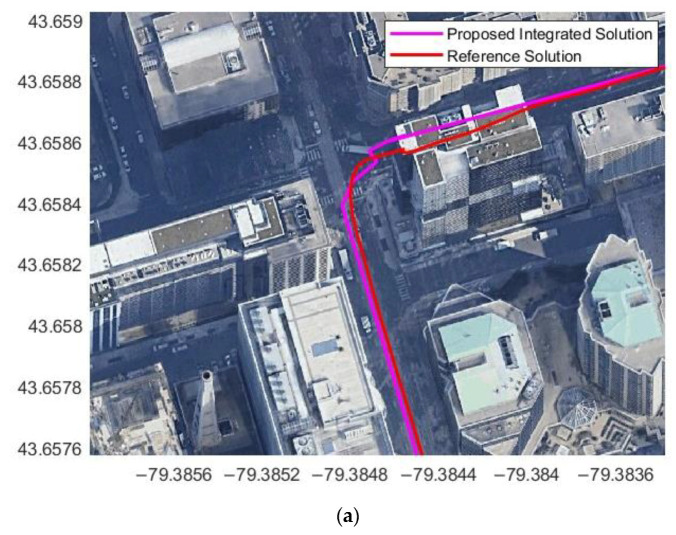

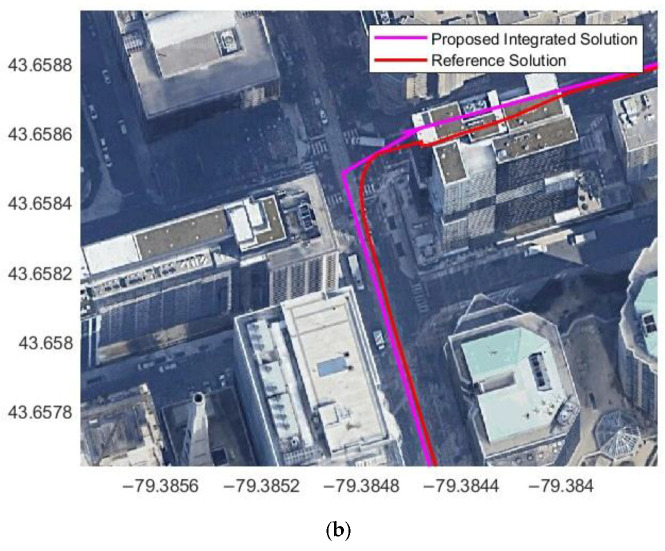
Radar/INS integrated solution at the intersection (**a**) with map matching and (**b**) without map matching.

The map-matching technique was applied during the GNSS outage. However, at the road intersections, the map-matching algorithm was disabled since the map lines intersected at 90° (straight lines) while the vehicle moved from one line to another line through a curve. Therefore, the map-matching technique was disabled during intersections and enabled when the vehicle left the intersection and moved in a straight line, as shown in Figure 15.

## 4. Conclusions

This research proposed a new technique based on radar/INS integration and map matching for robust vehicle navigation in GNSS-challenging environments. The proposed system consists of four static FMCW radar units and OSM for map matching. Two radar units were used to estimate the vehicle’s forward speed, while the four radar units were used to estimate the vehicle’s transition and rotation. The radar solution was first integrated with the INS solution, and then map-matching techniques were employed to correct the integrated position and obtain the final navigation solution. Road intersections were detected from the azimuth angular velocity measurements, and map matching was deactivated at the intersections. Real driving data were collected to test the proposed method, and simulated GNSS signal outages were introduced. The percentage RMSE error was less than 1% of the traveled distance in most cases during two and three minutes of GNSS signal outage. 

One of the limitations of the proposed algorithm is that the map accuracy will limit the final accuracy. Additionally, the OSM maps are open source so anyone can edit or update the map. Therefore, it is recommended to use high-definition maps to improve the corrected position accuracy. In future work, a comparison of the estimated forward velocity from the radar units with the forward velocity from the vehicle odometer will be investigated. Furthermore, the use of one EKF or different sensor fusion filters and architectures will be studied. Finally, a smart module will be developed to automatically switch from GNSS/INS integration to radar/INS integration based on factors such as the satellite geometry and the number of visible satellites.

## Figures and Tables

**Table 1:** Position RMSE from radar/INS integration with map matching during different simulated GNSS outage durations.

Outage Duration (s)	Mean Error (m)	RMSE (m)	Traveled Distance (m)	Percentage RMS Error (%)
30	2.22	2.69	259.34	1.04
60	7.56	8.40	599.70	1.40
90	13.25	22.89	929.82	2.46

**Table 2:** Position RMSE from radar/INS integration with map matching during different durations of a simulated GNSS signal outage.

Outage Duration (s)	Mean Error (m)	RMSE (m)	Traveled Distance (m)	Percentage RMS Error (%)
30	1.13	1.19	63.22	1.88
60	0.90	1.25	204.18	0.61
90	1.85	2.50	290.92	0.86
120	2.40	3.08	304.15	1.01
180	2.06	2.72	611.69	0.44

**Table 3:** Position RMSE from radar/INS integration with map matching during different simulated GNSS signal outage durations.

Outage Duration (s)	Mean Error (m)	RMSE (m)	Traveled Distance (m)	Percentage RMS Error (%)
30	3.08	3.49	211.54	1.65
60	3.35	3.51	495.16	0.71
90	3.15	3.35	524.50	0.64
120	3.45	4.11	679.49	0.61
180	3.88	4.47	845.74	0.53

## Data Availability

The data presented in this study is available on request from the corresponding author. The data are not publicly available due to its large size.

## References

[1] Rabbou M.A., El-Rabbany A. (2015). Integration of GPS Precise Point Positioning and MEMS-Based INS Using Unscented Particle Filter. Sensors.

[2] Chiang K.W., Duong T.T., Liao J.K. (2013). The Performance Analysis of a Real-Time Integrated INS/GPS Vehicle Navigation System with Abnormal GPS Measurement Elimination. Sensors.

[3] Iqbal U., Georgy J., Korenberg M.J., Noureldin A. Augmenting Kalman Filtering with Parallel Cascade Identification for Improved 2D Land Vehicle Navigation. Proceedings of the 2010 IEEE 72nd Vehicular Technology Conference-Fall.

[4] Al Bitar N., Gavrilov A.I. (2019). Comparative Analysis of Fusion Algorithms in a Loosely-Coupled Integrated Navigation System on the Basis of Real Data Processing. Gyroscopy Navig..

[5] Li T., Zhang H., Niu X., Gao Z. (2017). Tightly-Coupled Integration of Multi-GNSS Single-Frequency RTK and MEMS-IMU for Enhanced Positioning Performance. Sensors.

[6] Falco G., Pini M., Marucco G. (2017). Loose and Tight GNSS/INS Integrations: Comparison of Performance Assessed in Real Urban Scenarios. Sensors.

[7] Gao L., Xiong L., Xia X., Lu Y., Yu Z., Khajepour A. (2022). Improved Vehicle Localization Using On-Board Sensors and Vehicle Lateral Velocity. IEEE Sens. J..

[8] Gao Y., Liu S., Atia M.M., Noureldin A. (2015). INS/GPS/LiDAR Integrated Navigation System for Urban and Indoor Environments Using Hybrid Scan Matching Algorithm. Sensors.

[9] Zou Q., Sun Q., Chen L., Nie B., Li Q. (2022). A Comparative Analysis of LiDAR SLAM-Based Indoor Navigation for Autonomous Vehicles. IEEE Trans. Intell. Transp. Syst..

[10] Chang L., Niu X., Liu T., Tang J., Qian C. (2019). GNSS/INS/LiDAR-SLAM Integrated Navigation System Based on Graph Optimization. Remote Sens..

[11] Song K.-T., Chiu Y.-H., Kang L.-R., Song S.-H., Yang C.-A., Lu P.-C., Ou S.-Q. Navigation Control Design of a Mobile Robot by Integrating Obstacle Avoidance and LiDAR SLAM. Proceedings of the 2018 IEEE International Conference on Systems, Man, and Cybernetics (SMC).

[12] Hide C., Botterill T., Andreotti M. Low Cost Vision-Aided IMU for Pedestrian Navigation. Proceedings of the 2010 Ubiquitous Positioning Indoor Navigation and Location Based Service.

[13] Panahandeh G., Jansson M. (2014). Vision-Aided Inertial Navigation Based on Ground Plane Feature Detection. IEEE/ASME Trans. Mechatron..

[14] Borenstein J., Feng L. (1996). Measurement and Correction of Systematic Odometry Errors in Mobile Robots. IEEE Trans. Robot. Autom..

[15] Dissanayake G., Sukkarieh S., Nebot E., Durrant-Whyte H. (2001). The Aiding of a Low-Cost Strapdown Inertial Measurement Unit Using Vehicle Model Constraints for Land Vehicle Applications. IEEE Trans. Robot. Autom..

[16] Liu C.Y., Lin C.A., Chiang K.W., Huang S.C., Chang C.C., Cai J.M. Performance Evaluation of Real-Time MEMS INS/GPS Integration with ZUPT/ZIHR/NHC for Land Navigation. Proceedings of the 2012 12th International Conference on ITS Telecommunications, ITST 2012.

[17] Elkholy M., Elsheikh M., El-Sheimy N. Radar-Based Localization Using Visual Feature Matching. Proceedings of the 34th International Technical Meeting of the Satellite Division of the Institute of Navigation, ION GNSS+ 2021.

[18] Elkholy M., Elsheikh M., El-Sheimy N. Radar/IMU Integration Using Visual Feature Matching. Proceedings of the 35th International Technical Meeting of the Satellite Division of The Institute of Navigation (ION GNSS+ 2022).

[19] Elkholy M., Elsheikh M., El-Sheimy N. (2022). Radar/INS Integration for Pose Estimation in GNSS-Denied Environments. Int. Arch. Photogramm. Remote Sens. Spat. Inf. Sci..

[20] Rashed M.A., Abosekeen A., Ragab H., Noureldin A., Korenberg M.J. Leveraging FMCW-Radar for Autonomous Positioning Systems: Methodology and Application in Downtown Toronto. Proceedings of the 32nd International Technical Meeting of the Satellite Division of the Institute of Navigation, ION GNSS+ 2019.

[21] Abosekeen A., Noureldin A., Korenberg M.J. Utilizing the ACC-FMCW Radar for Land Vehicles Navigation. Proceedings of the 2018 IEEE/ION Position, Location and Navigation Symposium, PLANS 2018-Proceedings.

[22] Nießner M., Dai A., Fisher M. (2014). Combining Inertial Navigation and ICP for Real-Time 3D Surface Reconstruction. Eurographics (Short Pap.).

[23] Xu X., Luo M., Tan Z., Zhang M. Improved ICP Matching Algorithm Based on Laser Radar and IMU. Proceedings of the 2018 5th IEEE International Conference on Cloud Computing and Intelligence Systems (CCIS).

[24] Censi A. An ICP Variant Using a Point-to-Line Metric. Proceedings of the 2008 IEEE International Conference on Robotics and Automation.

[25] Biber P., Strasser W. The Normal Distributions Transform: A New Approach to Laser Scan Matching. Proceedings of the Proceedings 2003 IEEE/RSJ International Conference on Intelligent Robots and Systems (IROS 2003) (Cat. No.03CH37453).

[26] Levinson J., Thrun S. Robust Vehicle Localization in Urban Environments Using Probabilistic Maps. Proceedings of the 2010 IEEE International Conference on Robotics and Automation.

[27] Haklay M., Weber P. (2008). OpenStreetMap: User-Generated Street Maps. IEEE Pervasive Comput..

[28] Park Y., Kim J., Kim A. Radar Localization and Mapping for Indoor Disaster Environments via Multi-modal Registration to Prior LiDAR Map. Proceedings of the IEEE/RSJ International Conference on Intelligent Robots and Systems (IROS).

[29] Greenfeld J.S. (2002). Matching GPS Observations to Locations on a Digital Map. Transp. Res. Board.

[30] Scott C. (1994). Improved GPS Positioning for Motor Vehicles through Map Matching. Proc. ION GPS.

[31] Syed S., Cannon M.E. (2004). Fuzzy Logic Based-Map Matching Algorithm for Vehicle Navigation System in Urban Canyons. Proc. Natl. Tech. Meet. Inst. Navig..

[32] Liu W., Xia X., Xiong L., Lu Y., Gao L., Yu Z. (2021). Automated Vehicle Sideslip Angle Estimation Considering Signal Measurement Characteristic. IEEE Sens. J..

[33] Radar UMRR-11 Type 132 Data Sheet. https://autonomoustuff.com/products/sms-automotive-radar-umrr-11.

[34] Xsens MTi-G-710 Data Sheet. https://www.xsens.com/hubfs/Downloads/Leaflets/MTi-G-710.pdf.

[35] Radar UMRR-96 Type 153 Data Sheet. https://autonomoustuff.com/products/smartmicro-automotive-radar-umrr-96.

[36] u-blox ZED-F9R module-Data Sheet. https://www.u-blox.com/en/product/zed-f9r-module.

